# Emerging Roles of NDUFS8 Located in Mitochondrial Complex I in Different Diseases

**DOI:** 10.3390/molecules27248754

**Published:** 2022-12-09

**Authors:** Sifan Wang, Yuanbo Kang, Ruifeng Wang, Junqi Deng, Yupei Yu, Jun Yu, Junpu Wang

**Affiliations:** 1 Department of Pathology, Xiangya Hospital, Central South University, Changsha 410008, China; wangsifan199909@163.com (S.W.); kangyuanbo2000@163.com (Y.K.); wrf19962020@163.com (R.W.); 17707379182@163.com (J.D.); 18879486723@163.com (Y.Y.); 2 Department of Pathology, School of Basic Medicine, Central South University, Changsha 410008, China; 3 Xiangya School of Medicine, Central South University, Changsha 410013, China; 4 National Clinical Research Center for Geriatric Disorders, Xiangya Hospital, Central South University, Changsha 410008, China; 5 Department of Dermatology, Peking Union Medical College Hospital, Chinese Academy of Medical Sciences and Peking Union Medical College, Beijing 100730, China; 6 Department of Neurology, Third Xiangya Hospital, Central South University, Changsha 410008, China

**Keywords:** NDUFS8, mitochondrial complex I, Leigh syndrome, cancer, diabetes mellitus, metabolism

## Abstract

NADH:ubiquinone oxidoreductase core subunit S8 (NDUFS8) is an essential core subunit and component of the iron-sulfur (FeS) fragment of mitochondrial complex I directly involved in the electron transfer process and energy metabolism. Pathogenic variants of the *NDUFS8* are relevant to infantile-onset and severe diseases, including Leigh syndrome, cancer, and diabetes mellitus. With over 1000 nuclear genes potentially causing a mitochondrial disorder, the current diagnostic approach requires targeted molecular analysis, guided by a combination of clinical and biochemical features. Currently, there are only several studies on pathogenic variants of the *NDUFS8* in Leigh syndrome, and a lack of literature on its precise mechanism in cancer and diabetes mellitus exists. Therefore, NDUFS8-related diseases should be extensively explored and precisely diagnosed at the molecular level with the application of next-generation sequencing technologies. A more distinct comprehension will be needed to shed light on NDUFS8 and its related diseases for further research. In this review, a comprehensive summary of the current knowledge about NDUFS8 structural function, its pathogenic mutations in Leigh syndrome, as well as its underlying roles in cancer and diabetes mellitus is provided, offering potential pathogenesis, progress, and therapeutic target of different diseases. We also put forward some problems and solutions for the following investigations.

## 1. Introduction

Mitochondria in eukaryotes play a vital role in bioenergetics, metabolism, as well as signal transduction, and are related to various diseases [1]. Mitochondria contain at least 1500 different proteins in humans, functioning as core parts in energy metabolism, the fusion and fission of mitochondria, and the regulation of cell function [1,2]. From 20 to 25% of these proteins were used to express, regulate, and maintain the mitochondrial genome that codes for only 1% of mitochondrial proteins, and only up to 15% were directly involved in energy metabolism [3]. About 99% of mitochondrial proteins are encoded by nuclear genes, synthesized in the cytosol, and directed onto the membrane and then into the subcompartments of mitochondria by different signal pathways [4]. The invaginations of the mitochondrial inner membrane contain the respiratory complexes I to IV and the F_1_F_0_-ATP synthase, which constitute the oxidative phosphorylation system [1].

Mitochondrial complex I is also named mitochondrial NADH: ubiquinone oxidoreductase is the largest multi-subunit enzyme of the mitochondrial respiratory chain with 38 nuclear-encoded subunits and 7 subunits encoded by the mitochondrial genome [5]. Mitochondrial complex I, the initial and key limiting enzyme, can catalyze the transfer of electrons from Nicotinamide adenine dinucleotide (NADH) to ubiquinone through the respiratory chain, whose changes in the structure and function lead to wide varieties of clinical syndromes ranging from lethal encephalopathies to neurodegenerative disorders [6,7]. Complex I also consists of the I–III–IV supercomplexes, which may coordinate the activity of respiratory complexes, affect the assembly and stability of the complexes, and/or reduce the formation of reactive oxygen species (ROS) [8].

NADH:ubiquinone oxidoreductase core subunit S8 (NDUFS8), also called TYKY, is a nuclear-encoded subunit of human mitochondrial complex I with a molecular size of about 23 kDa. NDUFS8 is one of the core subunits of complex I, directly involved in the electron transfer process catalyzed by complex I owing to its special structure of containing tetranuclear FeS clusters (N6a and N6b) [9,10]. Based on this, pathogenic variants of the *NDUFS8* are relevant to infantile-onset and severe diseases, including Leigh syndrome, cancer, and diabetes mellitus [6,9]. The first nuclear-encoded subunit mutations of complex I in a patient with Leigh syndrome are missense mutations in the *NDUFS8*, followed by several patients with different pathogenic variants [11]. In addition, with the development of the link between other subunits of complex I and cancer or diabetes mellitus, the results have gradually emerged, while the relation between NDUFS8 and these diseases is poorly studied [12,13,14,15]. Therefore, we summarize the structure, function, and possible roles or mechanisms of NDUFS8 in diseases in this review, establishing the foundation for future research.

## 2. The Structures and Functions of Complex I and NDUFS8

**Figure 1:**
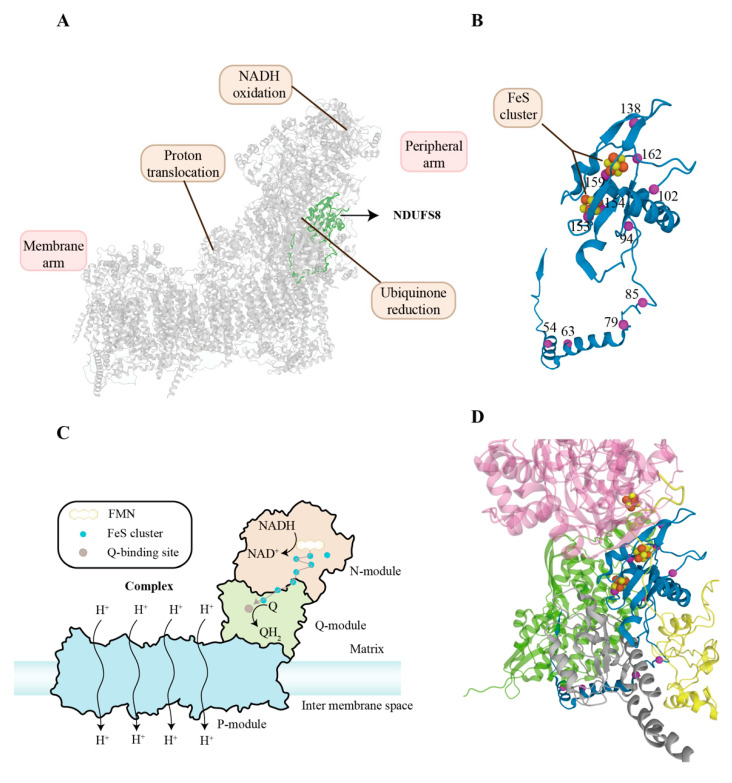
Location, structure, functional mechanism, and clinical pathogenic variants of NDUFS8. (**A**) Cryo-EM structure of human respiratory complex I (CI) with other subunits in grey from 5XTD (EMD-6773) [8]. The ribbon highlighted in green represents NDUFS8. (**B**) The folds of NDUFS8 are shown in ribbon representation. NDUFS8 contains two [4Fe–4S]^2+/1+^ clusters represented by a combination of red and yellow balls. The pathogenic mutation sites of *NDUFS8* are presented by magenta balls. (**C**) Schematic representation of components and functions of CI. CI consists of three functional modules: NADH oxidation (N module), ubiquinone reduction (Q module), and proton translocation (P module). They form an L-shape: one hydrophobic arm oriented parallel to the membrane and a hydrophilic arm extending into the mitochondrial matrix. There is a chain of seven FeS clusters that can transfer electrons from FMN to the ubiquinone reduction site. The P module harbors proton-translocation pathways connecting the mitochondrial matrix and the intermembrane space. (**D**) NDUFS8 (blue), NDUFS1 (pink), NDUFS2 (green), NDUFS7 (grey), and NDUFA12 (yellow) of complex I are shown. The annotated pathogenic mutation residues probably have effects on NDUFS8 and its surroundings, leading to complex I dysfunction [28]. Among them, p.Cys153Arg, p.Gly154Ser, and p.Val162Met are associated with the surroundings of two [4Fe–4S]^2+/1+^ clusters in NDUFS8, possibly disrupting electron transfer. The p.Ala159Asp lies next to Cys126, a component of the surroundings of two [4Fe–4S]^2+/1+^ clusters in NDUFS8. The p.Arg138His is linked to the disruption of electron transfer at the N5 cluster in NDUFS1. Moreover, the p.Arg94Cys is responsible for the impaired interaction of NDUFS8 and NDUFS2, while the p.Pro85Leu is associated with the impaired interaction of NDUFS8, NDUFA12, and NDUFS7. Abbreviations: NDUFS8: NADH:ubiquinone oxidoreductase core subunit S8; NDUFA12: NADH:ubiquinone oxidoreductase subunit A12; FMN: flavine mononucleotide; CI: complex I; NADH: Nicotinamide adenine dinucleotide; Q: ubiquinone.

As mentioned above, complex I (CI), a redox-driven proton pump, can transfer electrons from NADH to ubiquinone, and translocate vectorial proton across the inner mitochondrial membrane [16,17]. The structure of CI has been described by X-ray crystallography and cryo-EM [8,18,19,20,21,22] (Figure 1A). It is an L-shaped molecule containing two domains: a hydrophilic peripheral domain extending into the mitochondrial matrix and a hydrophobic membrane domain [23,24]. The hydrophilic arm comprises the NADH oxidation module (N-module), which provides electron input into the chain of [FeS] clusters, and the ubiquinone reduction module (Q-module), which conducts electrons to the ubiquinone-binding site. There is a chain of seven FeS clusters that can transfer electrons from flavin mononucleotide (FMN) to the ubiquinone reduction site [25]. The hydrophobic arm comprises the proton translocation module (P-module), which catalyzes proton transport (Figure 1C) [26,27].

NDUFS8 and homologues are highly conserved among eukaryotes and prokaryotes and extensively observed at various levels in all tissues, especially the heart and skeletal muscle [9,29,30,31,32,33]. The *NDUFS8* is located on chromosome 11q13 immediately downstream of the aldehyde dehydrogenase 7 isoform gene, splitting into seven exons and six introns [9]. Its cDNA contains an open reading frame of 633 bp, coding for 210 amino acids [11]. The general transcription factors nuclear respiratory factor 1/2 (NRF1/2), Specificity Protein 1 (Sp1), and Yin Yang 1 (YY1) can activate transcription of the *NDUFS8* [34]. As the electrical driving unit for a proton pump, NDUFS8 is a 23 kDa special ferredoxin and contains two [4Fe–4S]^2+/1+^ ferredoxin consensus patterns (N6a and N6b) (Figure 1B), which have long been thought to provide the binding site for the iron-sulfur ending cluster N-2 [35,36]. and there are two four-cysteine patterns (PROSITE pattern PS00198: CIACKLCEAICP and CIYCGFCQEACP) that ligand the two [4Fe–4S]^2+/1+^ clusters in NDUFS8 [34]. NDUFS8 is located within the ubiquinone-binding module (Q-module) involved in the early steps of respiratory complex I (CI) assembly, which implies its indispensable role in mitochondrial function [37]. A recent study has demonstrated in detail that NDUFS8 plays a crucial role in the global regulation of the cell growth and secondary metabolism of *Monascus*, revealing that its exact molecular mechanism is via changing the intracellular ROS and ATP levels [38].

## 3. Roles of NDUFS8 in Diseases

### 3.1. Pathogenic Variants of the NDUFS8 and Leigh Syndrome

Complex I deficiency caused by complex I gene mutation is highly relevant to mitochondrial disorders, leading to neuromuscular symptoms and various clinical manifestations [39]. Among them, Leigh syndrome (LS, OMIM: 256000) is the most common pediatric presentation of the mitochondrial disorder [40]. Typically first seen before 12 months of age, LS was first described by Denis Leigh in 1951 and characterized by focal, bilaterally symmetrical, and subacute necrotic lesions in the brainstem, the thalamus, and the posterior columns of the spinal cord [41]. Devastatingly, this neurodegenerative disorder is both phenotypically and genetically heterogeneous, and so far, pathogenic mutations in more than 75 genes have been proven, encoded by nuclear and mitochondrial genomes, especially the mutations referring to the mitochondrial respiratory enzyme complex or pyruvate dehydrogenase complex [40,42,43].

To date, mitochondrial complex I deficiency is the most common biochemical cause of LS, nearly constituting one-third of its etiology [40], and pathogenic variants of the *NDUFS8* can be responsible for LS, including homozygous (c.236C > T/p.Pro79Leu, c.460G > A/p.Gly154Ser, c.187G > C/p.Glu63Gln, c.160C > T/p.Arg54Trp, c.281C > T het/p.Arg94Cys) and compound heterozygous (c.236C > T/p.Pro79Leu and c.305G > A/p.Arg102His, c.254C > T/p.Pro85Leu and c.413G > A/p.Arg138His, c.229C > T/p.Arg77Trp and c.476C > A/p.Ala159Asp, c.52C > T/p.Arg18Cys, c.484G > T/p.Val162Met, c.457T > C/p.Cys153Arg) variations in NDUFS8 (Figure 1D) [6,44,45,46]. These pathogenic variations influence assembly of complex I not only because of the misfolding of NDUFS8 but also owing to impaired interaction of other subunits and NDUFS8 [28]. Among them, c.236C > T/p.Pro79Leu and c.305G > A/p.Arg102His along with c.364G > A in the *NDUFS7* gene have successfully been reconstructed by using the obligate aerobic yeast *Yarrowia lipolytica*, exhibiting similar complex I defects and possibly sharing their common characteristics in the pathogenesis of LS [47].

By searching the literature, 16 patients with Leigh syndrome carrying *NDUFS8* mutations and 1 patient with encephalomyopathy carrying *NDUFS8* mutations have been displayed in Table 1 below [48]. To date, all identified mutations were missense mutations, and no patients were reported with nonsense mutations in *NDUFS8*, which suggested that a complete absence of this protein might lead to intrauterine lethality [6]. The clinical manifestations of LS shown in Table 2 were intricate and manifold and varied from person to person [49]. Of the 13 patients, some showed severe manifestations with respiratory problems, feeding difficulties, epilepsy, and hypertrophic cardiomyopathy, and died within three months of life [11,50]. Others had normal or mildly impaired motor development in the first year of life and developed a slowly progressive neurological disease during childhood, such as slowly progressive muscle weakness, dysarthria, ataxic gait, and severe myopia [6,51]. According to a study in the Drosophila Model of LS, the severity of manifestations varied possibly depending on the maternally inherited mitochondrial background, and mitochondria–nuclear interactions affected lifespan and neurodegeneration [52]. In terms of genetic diagnosis of LS at present, the strategy is rapidly evolving as next-generation sequencing (NGS) technologies, and whole-exome sequencing (WGS), as well as whole-exon sequencing (WES), is becoming available [43,53]. This will make the molecular diagnosis of LS by screening patients’ genes possible [40].

Currently, symptomatic rather than etiological treatments are available for LS, which has been a challenge for researchers [58]. With the improvement of therapeutic technology, a cell-penetrating peptide derived from the HIV-1 trans-activator of transcription protein (TAT) has been successfully applied as a carrier to bring fusion proteins into cells without compromising the biological function of the cargoes. Therefore, Lin et al. successfully developed a TAT-mediated protein transduction system to rescue complex I deficiency caused by NDUFS8 defects and proved that treatment with TAT-NDUFS8 not only significantly ameliorated the assembly of complex I in a NDUFS8-deficient cell line but also partially rescued complex I function both in the in-gel activity assay and the oxygen consumption assay [10]. This study has provided us with a new approach to the treatment of Leigh syndrome.

### 3.2. NDUFS8 and Cancer

Functional mitochondria are essential for the cancer cell [59], and their dysfunction influences the balance of the intracellular environment through complicated signal pathways during cancer oncogenesis and progression [60]. Among all mitochondrial biochemical steps, complex I is the first entrance step of oxidative phosphorylation [61]. Complex I has also been recognized as one of the main sources of ROS, which is linked to cancer cell survival, proliferation, transformation, and malignancy progression [62,63,64,65]. Contrary to the increasing attention to mtDNA mutation in carcinogenesis [66,67,68], the roles of core nuclear-encoded subunits have not yet been much explored, such as NDUFS1, NDUFS2, NDUFS3, NDUFS7, NDUFS8, NDUFV1, and NDUFV2.

**Figure 2:**
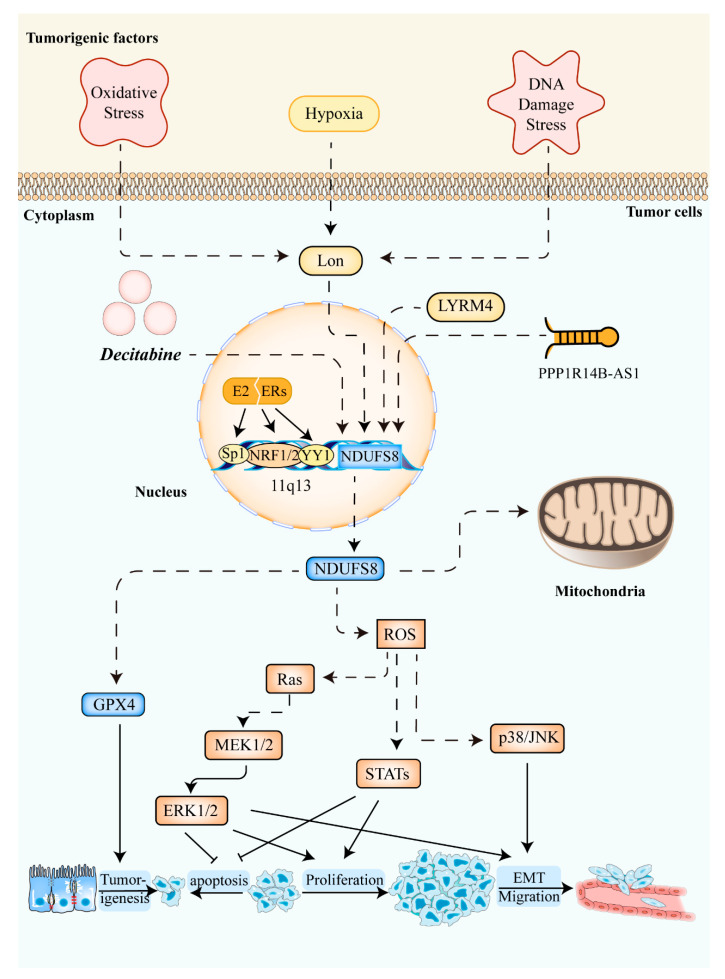
The roles of NDUFS8 in cancers. Several cancerogenic factors, such as oxidative stress, hypoxia, and DNA damage stress upregulate the level of mitochondrial Lon. Then, the elevated expression of NDUFS8 via the mitochondrial Lon caused an increased level of ROS in mitochondria [62]. Increased ROS concentration in tumor cells improves cell survival, proliferation, transformation, and migration through the activation of Ras-ERK1/2 signaling [78], STATs, and/or p38/JNK [62]. Meanwhile, the expression of NDUFS8 shows a strong positive correlation with GPX4 expression linked to tumorigenesis [79]. Moreover, induced by the E_2_/ER pathway [80], the general transcription factors NRF1/2, Sp1, and YY1 can activate the transcription of *NDUFS8*. LYRM4 [81], long non-coding RNA PPP1R14B-AS1 [82], and decitabine [83] can also increase NDUFS8 expression in some cancer cells. Abbreviations: NDUFS8: NADH:ubiquinone oxidoreductase core subunit S8; ROS: Reactive Oxygen Species; GPX4: Glutathione peroxidase 4; EMT: epithelial–mesenchymal transition; MEK1/2: mitogen-activated protein kinase kinases 1/2; ERK1/2: extracellular signal-regulated kinase 1/2; STATs: signal transducers and activators of transcription; JNK: c-Jun N-terminal kinase; E_2_: estrogen; ER: estrogen receptor; NRF1/2: nuclear respiratory factor 1/2; Sp1: Specificity Protein 1; YY1: Yin Yang 1; LYRM4: LYR (leucine/tyrosine/arginine) motif protein 4.

Among the 7 nuclear-encoded subunits above, the overexpression of *NDUFS1* could augment the activity of complex I, reverse glycolysis, and enhance the radiosensitivity of colorectal cancer cells in vivo and in vitro [69]. NDUFS2, which impedes anticancer immune surveillance, is the primary target of the antitumor compound SMIP004-7 [70]. Not only core subunits but also accessory subunits are highly associated with the biological function of cancer cells. NDUFAF5 is one of the potential targets of NMS-873, which has been verified to reduce drug resistance in colon cancer cells [71]. The decrease of micropeptide in mitochondria could promote hepatoma metastasis by enhancing complex I activity, which could be attenuated by knocking down *NDUFA7* [72]. Likewise, NDUFS8 is involved in carcinogenesis, tumor metabolism, and progression, as shown in Figure 2 [73]. For instance, owing to the upregulation of NDUFS8 mediated by mitochondrial Lon, their overexpression facilitated ROS generation, causing carcinogenesis and progression [62,74,75,76]. Meanwhile, deleterious mtDNA mutations were able to increase ROS, providing a proliferative advantage to cancer [77].

Studies verified that high *NDUFS8* and low *NDUFS1* expressions were correlated with poor prognosis in patients and played a leading prognostic role in non-small-cell lung cancer (NSCLC), suggesting that their expressions might predict lung cancer prognosis [73,84]. This result could be possibly explained due to their different locations in mitochondria. NDUFS8 is located within the Q-module involved in the early steps of respiratory complex I (CI) assembly, while NDUFS1 is located within the N-module incorporated in the last step [37]. In this frame, reduced expression of Q-module subunits might have a more severe impact on the CI function than the N-module [73,85]. Consistent with high NDUFS8 expressions in NSCLC linking significantly reduced overall survival, studies suggested using mitochondrial markers as companion diagnostics in NSCLC patients, which was considerable for treatment stratification and personalized medicine [86]. For instance, a study found that NDUFS8 was one of the targets for the treatment of cyclopamine tartrate in NSCLC [87].

The elevated NDUFS8 could also be found in hepatocellular carcinoma (HCC) [81], which was associated with the dedifferentiation of HCC [88]. Likewise, the rise of NDUFS8 in HCC was modulated by LYR (leucine/tyrosine/arginine) motif protein 4 [81], helping HCC cells avoid sorafenib and successfully survive [89]. A combination of lung adenocarcinoma and liver cancer showed that *NDUFS8* was overexpressed as well and positively related to long non-coding RNA PPP1R14B-AS1, promoting tumor cell proliferation and migration via the enhancement of mitochondrial respiration [82].

Similarly, according to the survival analysis, the increased expression level of *NDUFS8* was associated with poorer overall survival in patients with acute myeloid leukemia (AML). However, how this gene related to the susceptibility and pathogenesis of AML was not extensively examined [79]. Recently, a rare *NDUFS8* R2C complex I variant in the germline of two AML patients was identified as heterozygous, exhibiting a decreased basal and maximal oxygen consumption rate [90]. By testing AML blood samples, 47 genes were annotated within 500 Kb of the association signal and the sentinel variant is a significant expression quantitative trait locus for 12 of these, including *NDUFS8* (PBH = 1.69 × 10^−4^) [91].

In breast cells, persistent overstimulation of estrogens and estrogen receptors contributed to the increase of NDUFS8 and ROS, gradually developing estrogen carcinogenesis (Figure 2) [80,92]. Importantly, *NDUFS8* has been reported to have an association with tumor relapse in patients with estrogen receptor α-positive breast cancer [93]. Despite the pathogenesis described previously, compared to non-TNBC subtypes, NDUFS8 was downregulated in triple-negative breast cancer (TNBCs) with a reduction in mitochondrial respiratory capacity [94,95].

It has been reported that loss of heterozygosity at chromosome 11q13 existed in up to 70% to 100% of adrenocortical carcinoma (ACC) [96,97]. *NDUFS8* was significantly differentially expressed between benign and malignant adrenocortical tumors (*p* < 0.05) with an overall accuracy of 87–91%, suggesting that NDUFS8 was a good diagnostic marker for distinguishing ACC from adenoma [98]. Based on the underexpression of *NDUFS8* in ACC, decitabine, an inhibitor of DNA promoter methylation, recovered the expression of *NDUFS8*, which revealed a possible role of epigenetic gene silencing in adrenocortical carcinogenesis [83]. However, it was uncertain to confirm that NDUFS8 was an independent adverse predictor of shorter overall survival [99].

Besides, compared with the normal corresponding tissue, *NDUFS8* expression was upregulated in some tumors, such as non-functional pituitary adenoma [100], while its expression was significantly decreased in others, such as clear-cell renal cell carcinoma [101] and ovarian clear-cell carcinoma [102]. However, there were still other cancer models not showing the change in *NDUFS8* expression, possibly presenting that dysregulation of *NDUFS8* may be specific to a few kinds of cancers [83,88,103,104,105,106].

All in all, research on *NDUFS8* expression in cancers is mainly cell experiments and animal models, suggesting different expression levels and impacts in different cancer cells or even unworthiness, as shown in Table 3, and the main mechanism between cancer and NDUFS8 refers to mitochondrial dysfunction but lacks specific evidence [99]. Therefore, more extensive studies concerning NDUFS8 in different cancers need to be done with different approaches and aspects. For example, there are more than 50,000 sequenced and accumulated cancer genomes around the world by using WGS, WES, and RNA sequencing (RNA-Seq) [107,108,109]. When it comes to NDUFS8 and cancer, the techniques above, with a whole landscape of driver mutations [110], are of great importance to identify whether the *NDUFS8* is a structural variant and pathogen of cancer or only the changing levels of NDUFS8 is a symbol of mitochondrial dysfunction.

### 3.3. NDUFS8 and Diabetes Mellitus

Diabetes mellitus is one of the metabolic disorders, tightly associated with energy metabolism and mitochondrial function [111,112]. Some trials have been made in patients with diabetes mellitus, in which they discovered the possible role of NDUFS8 change. It has been found that a higher serum concentration of NDUFS8 was linked to higher insulin sensitivity among people with type 1 diabetes mellitus, which might reflect better mitochondrial function [113]. Conversely, the *NDUFS8* gene was highly expressed in the skeletal muscle tissue of patients with type 2 diabetes mellitus (T2DM), which might hint that upregulation of *NDUFS8* expression would affect the normal glucose metabolism of skeletal muscle tissue, causing insulin resistance and then promoting diabetes. This study also concluded that, through the bioinformatic analysis, NDUFS8 might be a potential therapeutic target, which needed further studies together with molecular experiments [114]. During the formation of T2DM, high glucose, as a strong metabolic stressor, also induced the upregulation of *NDUFS8* [115]. Additionally, NDUFS8 exhibited increased interaction with insulin receptor substrate 1, which was linked to inflammation-mediated insulin resistance in T2DM patients [116]. There was a similar result that showed that *NDUFS8* expression was upregulation in high-fat diet-induced obesity-dependent diabetes mouse models, especially in the liver tissue [117]. In other studies, some factors, such as Sirtuin 1, leucine, and berberine, could directly or indirectly regulate the expression of the *NDUFS8* and then change the secretory pathways and quantity of insulin, suggesting the agent role of NDUFS8 [118,119,120].

Since maternally inherited diabetes and deafness (MIDD) was first described [121], many studies on mitochondrial pathogenic variants have appeared successively. Currently, diabetes mellitus has also been reported in mitochondrial diseases caused by autosomal recessive mutations in the nuclear genes mtDNA polymerase gamma (1399G --> A), and mitochondrial inner membrane protein 17 (p.LysMet88-89MetLeu and p.Leu143 *) [122,123]. The m.14577T > C mutation in *MT-ND6*, which encodes NADH-ubiquinone oxidoreductase chain 6, a core subunit of complex I, was associated with diabetes mellitus [123]. When it comes to pathogenic variants of the *NDUFS8* and diabetes mellitus, there is no study available, which implies that we can use WGS and WES to explore if the mutations exist [124].

### 3.4. NDUFS8 and Other Diseases

Owing to the importance of NDUFS8 in metabolism, it has relations with different diseases. As a vital component of the mitochondrial electron transport chain, expressions of *NDUFS8* were detected or knocked out in many studies to reflect mitochondrial function and indicate the underlying mechanism [125,126,127,128,129]. For example, by measuring expressions of *NDUFS8*, microRNA-34a accelerated apoptosis of human lens epithelial cells via mitochondrial dysfunction [126]. Throughout the progression of sarcopenia, reduced NDUFS8 caused dysregulation of mitochondrial quality control in skeletal muscle in the senescence-accelerated mouse prone 8 [130]. The decreased NDUFS8 in sepsis was implicated in oxidative phosphorylation, showing disturbances in energy metabolism [131,132]. Embryos lacking NDUFS8 displayed the formation of an egg cylinder but lack of gastrulation features in mice, illustrating the initial value of NDUFS8 during the peri-gastrulation [133].

Besides, many substances can influence the kidney through NDUFS8. Paraoxonase1 deficiency and hyperhomocysteinemia changed the expression of mouse kidney proteins related to renal diseases, and downregulation of *NDUFS8* could account for the involvement of hyperhomocysteinemia and reduced Paraoxonase1 in kidney disease through energy metabolism [134,135]. In the kidney of lipopolysaccharide-treated animals, NDUFS8 decreased and then led to decreased fatty acids oxidation following lipopolysaccharide treatment [136]. Because of the mitochondrial structural disorder after acute kidney injury, the level of NDUFS8 was down about 50%, which was ameliorated by some materials, such as mitochondria-targeted antioxidant Mito-2,2,6,6-tetramethylpiperidine-N-oxyl by injectable self-assembling peptide hydrogel, a novel cyclic helix B peptide, 5-methoxyindole-2-carboxylic acid, and curcumin [137,138,139,140]. Interestingly, NDUFS8 played a role in the process that perfusion of isolated rat kidneys with mesenchymal stromal cells/extracellular vesicles prevents ischemic damage, creating a possible application of NDUFS8 in organ transplantation [141].

What’s more, a group studied the mitochondrial role in the development of doxorubicin-induced cardiotoxicity and tested a decrease of NDUFS8 in this process, which implicated a possible mechanism of doxorubicin-induced cardiotoxicity [142], and similarly, downregulation of the *NDUFS8* expression was also involved in the mechanism of Propofol-induced cytotoxicity in human-induced pluripotent stem cell-derived cardiomyocytes [143].

Moreover, NDUFS8 can have a relation with neural and psychiatric diseases. Studies showed that chronic stress affected the mitochondrial respiratory chain, and mitochondrial dysfunction might be a potential mechanism of psychiatric pathophysiology [144,145]. Compared with middle-aged individuals, there were obvious upexpressions of *NDUFS8* in the frontal cortex in those with Parkinson’s disease, but notable downexpressions in those with Parkinson’s disease with dementia [146]. With the measurement of a significant upregulation of *NDUFS8* in the resilient group compared with controls in rat models, it could be speculated that the regulation of this gene protected against stress [147]. Additionally, there was an appealing study about an antioxidant fruit named *Euterpe oleracea* which improved the expression of *NDUFS8* to recover rotenone-caused mitochondrial dysfunction in neuronal-like cells SH-SY5Y [148].

## 4. Conclusions and Prospects

NDUFS8 is a highly conserved multi-functional subunit of mitochondrial complex I, involved in the electron transfer process and energy production. During mitochondrial respiration, electron transfer through this protein is linked to ROS production, activating the downstream signal pathways and then making much difference to multiple cellular activities. Owing to its vital structure of tetranuclear FeS clusters (N6a and N6b) and function of electron transfer, NDUFS8 plays a crucial role in different diseases and clinical processes, although there is no exact mechanism for cancer and diabetes mellitus.

Since Denis Leigh’s first description of Leigh syndrome in 1951 [41], many pathogenic mutations causing Leigh syndrome have been revealed, mostly linked to pathways of energy generation, but only 14 pathogenic variants of *NDUFS8* causing Leigh syndrome have been reported. Pathogenic variants of *NDUFS8* contribute to the onset of Leigh syndrome primarily by affecting the assembly of complex I, not only because of the misfolding of NDUFS8, but also owing to impaired interaction of other subunits and NDUFS8 [28]. On account of no effective treatment for Leigh syndrome, therapeutic intervention can benefit several biochemical and genetic forms of Leigh syndrome. Consequently, determining the molecular basis should be taken into great account, indicating applications of WGS and WES and enabling the progress of novel therapeutics.

Mitochondrial function plays an essential role in cancer cells. We have recognized mutations of mtDNA in cancer cells for more than two decades [149]. General attention paid to mitochondrial nuclear-encoded gene mutations has greatly emerged since the defects of fumarate hydratase, succinate dehydrogenase, isocitrate dehydrogenase 1 (*IDH1*), and *IDH2* in cancer cells were established [150]. Currently, studies have demonstrated that tumor growth requires an electron transport chain (ETC) whose inhibition has an anti-tumor effect together with targeted treatment [151,152]. Under this circumstance, it is of vital significance to explore the proteins and genes of ETC. Therefore, we summarize the relations between cancer and NDUFS8 of complex I. On the one hand, the level of NDUFS8 shows a prognostic factor for NSCLC and AML in a combination of other subunits in complex I, possibly pointing out a therapeutic target for cancer. On the other hand, the expressions of *NDUFS8* vary from cancer to cancer, showing its specific roles in different cancer cells. However, most of these studies have focused on the expressions of *NDUFS8* rather than the genetic level, which lacks molecular mechanisms of oncogenesis, tumor formation, and progression. RNA interference techniques can be utilized to manipulate the expression of disease-related genes, including small interfering RNA (siRNA) and short hairpin RNA (shRNA) [153,154], which inspires people to develop novel methods of disease treatment [155]. Providing an efficient and accurate technology, CRISPR/Cas9 and modified versions have become a powerful tool for etiological analysis, drug development, and cell therapy through altering the genomes [156]. Therefore, the use of siRNA, shRNA, and CRISPR/Cas9 technology could be applied in the future research of NDUFS8-related disease modeling, diagnosis, and treatment.

As a complex metabolic disorder, diabetes mellitus has an impact on many tissues where mitochondrial dysfunction can also be observed. The interactions of diabetes and mitochondria have been under discussion, especially mitochondrial roles in the pathology of diabetes complications [157]. Based on such a situation, we concentrate on NDUFS8 and its potential influence on diabetes. According to recent studies, different types of diabetes mellitus show diverse relevance to NDUFS8, from reflecting mitochondrial function to promoting diabetes formation. Nevertheless, there is no published literature on pathogenic variants of *NDUFS8* in diabetes but pathogenic mutations exist in other mitochondrial subunits in diabetes. To further study their links, proteomic and genetic data should be carried out.

Besides Leigh syndrome, cancer, and diabetes mellitus, as a core subunit of complex I, NDUFS8 has a relation with neural and psychiatric diseases. However, when it comes to mitochondrial diseases, which are some of the most common inherited neurometabolic disorders, the involvement of NDUFS8 should not be overlooked, which might help understand the elucidation of mitochondrial disease mechanisms, gene therapies, and new drugs [158].

Regardless of the upregulation or downregulation of *NDUFS8*, the change may contribute to the metabolic disorder of mitochondria, causing related diseases. Although there are many diseases related to NDUFS8, the unknown significance of some pathogenic variants in *NDUFS8* remained, such as the variants of exertional rhabdomyolysis in *NDUFS8* [159]. Studies wholly on NDUFS8 are rare and there are mainly research works referring to NDUFS8 that lack more specific data and principally treat NDUFS8 as an agent for their mechanism. Further studies are required to determine the accurate mechanism between NDUFS8 and its related diseases and the potential value for prevention, diagnosis, prognosis, and therapeutics.

## Figures and Tables

**Table 1:** Patients carrying pathogenic variants of the *NDUFS8*.

Sex	Diagnosis	CI Activity	Variations of Nucleotide	Amino Acid	Proposed Reasons for Complex I Dysfunction	Reference
M	LS	↓ (69% of normal fibroblasts, 39% of normal muscle)	c.236C > T het, c.305G > A het	p.Pro79Leu, p.Arg102His	Disruption of electron transfer at N5 cluster site; stabilizes loop coordinating cluster N5	[11]
F	LS	↓ (43% of normal lymphoblasts, 31% of normal muscle)	c.254C > T het, c.413G > A het	p.Pro85Leu, p.Arg138His	Affected assembly due to impaired interaction of NDUFS8, NDUFA12, and NDUFS7; located at the loop interacting with the NDUFA12 and NDUFS7. Disruption of electron transfer at N5 cluster site; stabilizes another loop coordinating cluster N5	[28,51]
M	LS	n.d.	c.52C > T het	p.Arg18Cys	n.d.	[48]
M	n.d.	↓ (18% of normal fibroblasts)	c.281C > T hom	p.Arg94Cys	Affected assembly due to impaired interaction of NDUFS8 and NDUFS2; found in a loop making the interface with the NDUFS2	[54]
F	LS	↓ (31% of normal muscle)	c.236C > T hom	p.Pro79Leu	Affected assembly due to misfolding of NDUFS8; located close to a stabilizing amphipathic α helix	[50]
M	ME	n.d.	c.460G > A hom	p.Gly154Ser	n.d.	[55]
M	ME, HCM	↓ (52% of normal fibroblasts, 38% of normal muscle)	c.229C > T het, c.476C > A het	p.Arg77Trp, p.Ala159Asp	Affected assembly due to misfolding of NDUFS8; located close to a stabilizing amphipathicα helix. Disruption of electron transfer at N6a cluster site; placed next to Cys126 coordinating cluster N6a. The mutation will likely decrease the redox potential of N6a	[56]
M	LS	↓ (54% of normal fibroblasts, 8% of normal muscle)	c.187G > C hom	p.Glu63Gln	Affected assembly due to misfolding of NDUFS8; located at a stabilizing amphipathic α helix	[56]
F (the sister of the eighth)	LS	n.d.	c.187G > C hom	p.Glu63Gln		[56]
M *	LS	↓ (25% of normal muscle)	c.160C > T hom	p.Arg54Trp	Affected assembly due to misfolding of NDUFS8; located at a stabilizing amphipathic α helix	[6]
M *	LS	n.d.	c.160C > T hom	p.Arg54Trp		[6]
F *	LS	n.d.	c.160C > T hom	p.Arg54Trp		[6]
M	MC	n.d.	compound heterozygous	n.d.	n.d.	[44]
M	LS	n.d.	c.484G > A het	p.Val162Met	n.d.	[46]
F	LS	n.d.	c.484G > A het	p.Val162Met	n.d.	[46]
F	LS	n.d.	c.305G > A het	p.Arg102His	n.d.	[45]
F	LS	n.d.	c.457T > C het	p.Cys153Arg	n.d.	[45]

Table legends: “↓”: decrease; n.d.: not determined; M: male; F: female; LS: Leigh syndrome; ME: mitochondrial encephalopathy; HCM: hypertrophic cardiomyopathy; MC: mitochondrial cytopathy; CI activity: the proportion of complex I activity in patients with Leigh syndrome to control groups; het: heterozygous; hom: homozygous; NDUFA12: NADH dehydrogenase (ubiquinone) 1 alpha subcomplex 12; NDUFS7: NADH:ubiquinone oxidoreductase core subunit S7; NDUFS2: NADH:ubiquinone oxidoreductase core subunit S7; N5: one of the FeS clusters in NDUFS1; N6a: one of the two FeS clusters in NDUFS8. * The three patients from Marina et al. [6] are all the children of the consanguineous Afghan parents ordered by age.

**Table 2:** Clinical manifestations of Leigh syndrome.

Body System	Relative Clinical Manifestations	Reference
Pulmonary	Abnormal respiration, hypoxia, and respiratory failure	[11]
Cardiac	Hypertrophic cardiomyopathy, asymmetric septal hypertrophy, and ventricular septal defects	[56]
Gastrointestinal	Dysmotility, constipation, acid reflux, vomiting, anorexia, dysphagia, malnutrition, and failure to thrive	[57]
Urinary	Tubulopathy, nephrotic syndrome, and Fanconi syndrome	[57]
Neurologic	Seizures, cognitive impairment, hypotonia, dystonia, neuropathy, generalized weakness, hyporeflexia, ataxia, chorea, spasticity, central apnea, stroke (metabolic), and tremor	[50]
Metabolic/Endocrine	Lactic acidosis, diabetes mellitus, and thyroid dysfunction	[57]
Dermatologic	Abnormal odor of skin, hyperpigmented skin eruptions, and hypertrichosis	[57]
Musculoskeletal	Muscle weakness, ptosis, and pes cavus	[6]
Immunologic	Impaired immunity	[57]
Audiologic	Sensorineural hearing loss and auditory neuropathy	[57]
Ophthalmologic	Nystagmus, ophthalmoparesis, retinitis pigmentosa, and failure to acquire smooth pursuit	[6]

**Table 3:** *NDUFS8* expression in different types of tumors.

Cancer Type	*NDUFS8* Expression	References
Non-small-cell lung cancer	Up	[73]
Hepatocellular carcinoma	Up	[81]
Acute myeloid leukemia	Up	[79]
Triple-negative breast cancer	Down	[94]
Adrenocortical carcinoma	Down	[98]
Clear-cell renal cell carcinoma	Down	[101]
Ovarian clear-cell carcinoma	Down	[102]

## Data Availability

Not applicable.
